# Editorial: The role of glial cells in the pathophysiology and treatment of Alzheimer's disease

**DOI:** 10.3389/fnins.2025.1749170

**Published:** 2026-01-13

**Authors:** Simone M. Crivelli, Zainuddin Quadri, Daan van Kruining, Pilar Martinez-Martinez, Erhard Bieberich, Jean-Yves Chatton

**Affiliations:** 1 Department of Fundamental Neurosciences, University of Lausanne, Lausanne, Switzerland; 2 Department of Physiology, University of Kentucky, Lexington, KY, United States; 3 Department of Psychiatry and Neuropsychology, School for Mental Health and Neuroscience, Maastricht University, Maastricht, Netherlands

**Keywords:** Alzheimer's disease, astrocytes, microglia, ApoE, metabolism, inflammation

For decades, Alzheimer's disease (AD) was mainly studied from a neuron-centric perspective. However, growing evidence shows that dysfunctional glial cells can trigger and worsen neuronal damage. This Research Topic proposes a shift in how we view glial cells, not just as responders to neuronal injury, but as active players that can initiate, amplify, and sustain key AD pathologies when impaired. By bringing together diverse contributions spanning discovery of glial subtypes, mechanistic insights, and therapeutic approaches, this Research Topic explores how glial cells regulate inflammation, neuroprotection, protein aggregate clearance, and synaptic function; and how these processes can be targeted to influence disease progression.

The goal of this Research Topic was two-fold: first, to consolidate current knowledge on the roles of astrocytes and microglia across four key domains in AD, namely, inflammatory signaling, trophic and metabolic support, aggregate clearance, and synaptic homeostasis; and second, to identify therapeutic strategies that restore glial balance, rather than focusing solely on neurons. Below, we summarize the eight manuscripts included in this Research Topic, grouped by thematic focus.

**From mapping the research landscape on astrocyte function in AD to mechanistic insights**. Two bibliometric studies provide a quantitative overview of the field's growing focus on glia. One study, centered on glial fibrillary acidic protein (GFAP), highlights increasing interest in astrocyte reactivity, with persistent hotspots in oxidative stress and neuroinflammation, and rising attention to blood-based GFAP as a scalable biomarker for AD screening (Zou et al.). A complementary “top-100” analysis of astrocyte-related AD studies reveals a shift from descriptive to mechanistic themes, such as “activation,” “Aβ,” and pathway-level analysis, while identifying key institutions and researchers driving this change (He et al.). Together, these studies show a field increasingly focused on testable mechanisms and clinically relevant markers of glial dysfunction.

**Glial failure at the intersection of genes and metabolism**. Mechanistic studies in this Research Topic converge on two upstream regulators of glial function: lipid/genetic risk and cellular energy balance. One study using ApoE-knockout mice shows that ApoE deficiency disrupts microglial and astrocytic homeostasis in an age-dependent manner, increasing inflammation and impairing Aβ clearance in both brain and retina. Diet worsens these effects, and tear-based miRNAs emerge as potential non-invasive markers of glial stress (Wijesinghe et al.). A focused review on mitochondrial uncoupling protein 4 (UCP4) places astrocyte bioenergetics at the forefront: reduced UCP4 in AD, and evidence that boosting astrocyte-specific UCP4 restores metabolic balance and protects neuronal structure, suggest mitochondrial uncoupling as a promising therapeutic target (Crivelli et al.).

**Microglial mechanosensing reframes plaque biology**. Beyond chemical signals, microglia also respond to the physical environment of the AD brain. A state-of-the-art review of the Piezo1 mechanosensitive channel shows how microglia migrate toward the stiffer regions around amyloid plaques and translate mechanical cues into calcium-driven responses that affect phagocytosis, cytokine release, and plaque burden (Ikiz et al.). Notably, Piezo1 can be modulated by small molecules and lifestyle factors (e.g., fatty acids, exercise, ultrasound), expanding the therapeutic toolkit beyond traditional receptor-based approaches. Mechanosensing thus adds a new layer to our understanding of microglial control over aggregate clearance and inflammation.

**Gut–glia axes: peripheral levers for central immunity**. Two reviews explore how the microbiota–gut–brain axis influences glial states in AD. Dysbiosis alters microglial activation and astrocyte function through microbial metabolites (e.g., short-chain fatty acids) and pathogen-associated signals (e.g., lipopolysaccharide), affecting NF-κB/TLR4 signaling, oxidative stress, and synaptic pruning. The therapeutic implication is clear: prebiotics, probiotics, and post-biotics may help shift glial cells toward homeostatic states, reducing neuroinflammation and improving clearance and metabolic support (Wu and Wei, Patricio-Martínez et al.).

**Toward glia-centric interventions**. Translational studies in this Research Topic show how glial pathways can be targeted therapeutically. A comprehensive review of acupuncture in AD reports consistent modulation of microglial phenotypes: from pro-inflammatory to reparative via TLR4/NF-κB, NLRP3 inflammasome, and TREM2 signaling, with additional effects on complement-mediated synaptic pruning (Liu et al.). The Piezo1 framework suggests mechanotherapeutic and bioelectric strategies to enhance microglial phagocytosis (Ikiz et al.), while the UCP4 perspective supports astrocyte-targeted metabolic interventions to restore trophic support and redox balance (Crivelli et al.).

The eight articles included in this Research Topic collectively foster a glia-centric understanding of AD. In summary: bibliometric mapping shows where the field is investing; mechanistic studies reveal how lipid genetics and mitochondrial metabolism destabilize glial function; biophysical and peripheral perspectives (Piezo1 and the gut-brain axis) broaden the therapeutic landscape; and translational examples demonstrate that reprogramming glia is both feasible and promising.

